# Host metabolite production and microbiome dynamics: effects of long-term diatom adaptation to warming

**DOI:** 10.1093/ismeco/ycaf103

**Published:** 2025-06-19

**Authors:** Susana Agusti, Afrah Alothman, Peng Jin, Clara S Stanschewski, Rubén Díaz-Rúa, Abdul-Hamid Emwas, Upendra Singh, Mariusz Jaremko

**Affiliations:** King Abdullah University of Science and Technology, Biological and Environmental Science and Engineering Division, Marine Science Program, Thuwal 23955-6900, Makka, Saudi Arabia; King Abdullah University of Science and Technology, Biological and Environmental Science and Engineering Division, Marine Science Program, Thuwal 23955-6900, Makka, Saudi Arabia; School of Environmental Science and Engineering, Guangzhou University, Guangzhou 510006, China; King Abdullah University of Science and Technology, Biological and Environmental Science and Engineering Division, Marine Science Program, Thuwal 23955-6900, Makka, Saudi Arabia; King Abdullah University of Science and Technology Center for Desert Agriculture, Thuwal 23955-6900, Makka, Saudi Arabia; King Abdullah University of Science and Technology, Biological and Environmental Science and Engineering Division, Marine Science Program, Thuwal 23955-6900, Makka, Saudi Arabia; King Abdullah University of Science and Technology Core Labs, Thuwal 23955-6900, Makka, Saudi Arabia; King Abdullah University of Science and Technology Biological and Environmental and Science and Engineering, Bioscience Program, Thuwal 23955-6900, Makka, Saudi Arabia; King Abdullah University of Science and Technology Biological and Environmental and Science and Engineering, Bioscience Program, Thuwal 23955-6900, Makka, Saudi Arabia

**Keywords:** microbiome, ocean warming, long-term adaptation, richness, species interaction, metabolomics, diatom, mutualism, coevolution, Chaetoceros tenuissimus

## Abstract

Marine diatoms contribute significantly to global oceanic primary production, constituting ~25% of earth production but are susceptible to the impacts of ocean warming. While organisms’ adaptation to rising temperatures may mitigate these impacts, it could also disrupt species interactions, including those between hosts and their microbiomes. In our study, we examined thermal performance and diversity changes in the microbiome of the tropical diatom *Chaetoceros tenuissimus* after long-term adaptation to ambient (LA) and warming (LW) temperatures. We observed notable shifts in the metabolomic profile, with amino acids accumulating after LW adaptation and sugars accumulating after LA, while lipids content remained unchanged. After LW, the microbiota increased maximum growth rate without changing its temperature optimum. *Roseovarius* sp. dominated the microbiome community at both LA (49.6%) and LW strains (42.8%) proving a strong partnership. Extinctions were highest under warming occurring in the low-abundant genera, but different partners developed increasing richness, with changes induced in the core microbiome. Short-term warming, however, resulted in decreased richness. Beta diversity was associated with long-term adaptation instead of assay temperature. Our findings align with general plant species association models, suggesting that long-term evolution of mutualisms enhances diversity and strength, particularly under warming in the marine partnership studied here. Our experimental results highlight the importance of examine long-term coevolution of host- microbiome partnerships to improve our understanding of consequences of warming.

## Introduction

There is growing evidence that microalgae, the major oceanic primary producers, are able to adapt to global change drivers, such as elevated CO_2_ [1, 2], pollutants [3], and increased temperature [4, 5]. The short generation times and high population densities of microalgae confer a substantial capacity for rapid evolutionary responses to environmental changes [6]. Diatomic algae are an important component of phytoplanktonic communities representing the 40% of oceanic production [7, 8], with a relevant role in the biological carbon pump even in oligotrophic tropical waters [9]. Tropical diatoms could adapt quickly to ongoing warming, even while experiencing thermal extremes, by employing differing optimality models [5]. However, the adaptation of organisms to environmental changes including warming may alter species interactions, which are rarely considered in adaptation studies [10]. Microalgae and bacteria have co-occurred in oceans for millions of years, resulting in several types of interactions, ranging from mutualism to parasitism [11, 12]. The survival of these microbiome partnerships in a changing ocean will depend on co-evolutionary responses, i.e. reciprocal changes between interacting species [13].

It has been reported that antagonistic relationships acted as a driver for diversification and divergence of species [14, 15]. However, in a mutualistic relationship, the pair have a close ecological relationship and can act as agents of natural selection for each other. Asymmetric mutualism may, however, enhance long-term coexistence and facilitate biodiversity maintenance [10]. Most microbiome host relationships are mutualistic. In a mutualistic interaction, for instance, cocultured sulfate-reducing bacteria and methanogenic archaea cooperated to perform an energy-yielding reaction; their communities stabilized and showed higher growth rates and yields after hundreds of generations [16]. However, ocean warming and other stressors related to global change could result in mutualism breakdowns [17]. For insects, warming has reportedly disrupted interactions between hosts and symbionts as well as those between hosts and parasitoids [18]. High temperatures can also eliminate certain essential microorganisms’ partners, as has been described for both corals [19] and ants [20]. Moreover, at low latitudes, global change is driving the development of unique environmental conditions as temperatures surpass those seen over the course of most organisms’ recent evolution. These changes pose crucial questions regarding an organism’s range of evolutionary responses that need to be tackled [6, 17]. It is also critical to understand the persistence of partnerships in such unique conditions.

Warming could modify metabolites production in microalgae and therefore affecting the interaction between phytoplankton and their co-associated bacteria. Metabolomics studies have been widely used in a variety of fields to analyze the changes in biochemical mechanisms and metabolisms pathways against stress environmental factors such as heavy metals, chemical toxicity, and stressful pH and temperature conditions [21–23]. Microalgae metabolic profiling can be identified and quantified using numbers of advanced metabolomics robust tools such as nuclear magnetic resonance (NMR) spectroscopy and combined with or separated setup of gas chromatography coupled with mass spectrometry (GC–MS) [21, 24]. These robust metabolomics platforms provide a reproductive, an easy and fast identification and quantification of huge numbers of metabolites with less sample preparation steps and small sample concentrations [25]. According to recent metabolomics and transcriptomics studies on the diatom *Phaeodactylum tricornutum*, warm temperature enhanced the concentration of 2-oxoglutarate; the center of nitrogen and carbon metabolisms, therefore increased the fatty acid metabolism, glutamine and glutamate production, the urea cycle, and the tricarboxylic acid (TCA) cycle [26]. Diatoms and other microalgae coexist with bacteria in the oceans which can be both actively involved in complicated association that can greatly affect each other’s metabolism activities and therefore impact biogeochemical cycles [27].

In this study, we applied a long-term co-evolutionary approach [28] to investigate how adaptation to ocean warming affects the microbiome association and diversity of the diatom *Chaetoceros tenuissimus*, isolated from the warm subtropical Red Sea. Microalgal strains cultured in the lab form a community of associated bacteria despite axenic handling. These microcosms represent an asymmetric nutritional mutualism, where conditions favor microalgal growth while heterotrophic bacteria benefit from organic products released by the algae [29]. Over ~2 years, we exposed the diatom and its bacterial partners to two temperatures: (i) ambient 26°C (mean surface temperature of the Red Sea from 1982–2015) [30], and (ii) 30°C, representing projected sea surface temperature increases by 2100 under the high-emission scenario SSP5–8.5 (IPCC 2021). Previously, we found that *C. tenuissimus* adapted to long-term warming (LW) by increasing its thermal optimum and maximum growth rate, following a “hotter is better” strategy [5]. Here, we examined the thermal performance and metabolite changes in the bacterial microbiome coevolved with *C. tenuissimus* under both ambient and warming conditions. Using gas chromatography–mass spectrometry (GC–MS, Agilent) and nuclear magnetic resonance (NMR, 800 MHz, Bruker), we analyzed metabolite changes among ambient and warming-adapted diatom strains. We also assessed shifts in the bacterial community, noting extinctions and the emergence of new genera associated with each temperature. To test stability, we conducted a transplant experiment, moving the warmed consortium back to ambient conditions. Our findings were compared with the prediction that long-term evolution of asymmetric mutualistic associations strengthens mutualism and increases community richness.

## Methods and materials

### Culture conditions

The diatom species *C. tenuissimus* was isolated from coastal Red Sea waters using micropipettes and a micromanipulator system (Eppendorf TransferMan 4r, Germany) under an inverted microscope (Zeiss AxioObserver.Z1, Germany), allowing for the collection of both the cells and their surrounding microbial environment. *C. tenuissimus* was maintained as batch cultures growing in F/4 medium for ~2 years under ambient (26°C, the mean sea surface temperature of the Red Sea during 1982–2015) and warming conditions (30°C, high-warming emission scenario RCP SSP5-8.5 projected for the turn of the next century) [5]. After 2 years, the diatom *C. tenuissimus* had undergone ~2224 and 2280 generations under long-term ambient (LA) and long term-warming (LW) temperature selection, respectively. The thermal responses of *C. tenuissimus* bacteria microbiome growing under LA and LW conditions for ~2 years were determined by triplicate at nine assay temperatures. At the end of the long-term selection period, cultures under LA and LW conditions were inoculated into 200 ml flasks at an initial phytoplankton cell density of 3 × 10^5^ cells ml^−1^, and then incubated at 16, 18, 22, 24, 26, 30, 34, 36, and 38°C. The cultures grew with a light: dark cycle of 12: 12 h under 400 μmol photons m^−2^ s^−1^ for 8–12 days until the stationary phase was reached. After a 1-day acclimation period in the new culture medium and temperature, bacterial cell counts were performed every 24 h with 200–400 μl samples using a BD FACSCanto™ II flow cytometer (BD Bioscience, Oxford, UK) calibrated with CS&T beads. To each sample, 4 μm of SYBR Green I solution was added, after which samples were left in the dark for ~10 min to stain bacterial DNA, allowing for the examination of bacteria. The bacterial cells were distinguished from noise and other components of the sample based on their red and green fluorescence signals as well as the 90° side light scatter (SSC) signals. Bacterial growth rates at each assay temperature were calculated as the slope of the linear regression of ln (cell concentration) as a function of experimental day in the exponential growth phase. Cell abundance of the diatom *C. tenuissimus* was quantified in parallel with bacterial analyses by examining the samples under an optical microscope (LEICA DMI 3000B, Germany) using a hemocytometer.

The thermal reaction norms of *C. tenuissimus* and the bacteria that evolved under the LA and LW temperatures were assessed by applying the equation described by [31] and [32]:


(1) \begin{equation*} \boldsymbol{f}\left(\boldsymbol{T}\right)=\boldsymbol{a}{\boldsymbol{e}}^{\boldsymbol{bT}}\left[\mathbf{1}-{\left(\frac{\boldsymbol{T}-\boldsymbol{z}}{\boldsymbol{w}/\mathbf{2}}\right)}^{\mathbf{2}}\right], \end{equation*}

where the specific growth rate, *f*, depends on the temperature, *T*, and is defined as a function of the parameters *z*, *w*, *a,* and *b*. *w* is the temperature niche width (the range of temperatures over which the growth rate is positive), while *z*, *a*, and *b* possess no explicit biological meaning but interact to influence the rate of increase in growth with temperature, the maximum growth rate, and the optimum temperature for growth. Specifically, *z* determines the location of the maximum of the quadratic portion of this function. *a* and *b* are the Eppley curve coefficient and Eppley curve exponent, respectively. We estimated the critical thermal minimum (*CT_min_*, the lowest temperature at which the growth of bacteria is zero), critical thermal maximum (*CT_max_*, the highest temperature at which the growth of bacteria is zero), maximum growth rate (μ_max_), optimal temperature for growth (*T*_opt_, the temperature at which the growth rate is maximal), and thermal breadth (expressed as *B_80_*, 80% performance of the maximum growth rate breadth) [33] by numerically maximizing the equation after estimating the parameter values for each replicate. A Student’s *t*-test was used to test the differences in *CT_min_*, *CT_max_*, *T*_opt_, μ_max_, and *B_80_* between the two temperature-adapted populations.

### Metabolites extraction and identification

For metabolites extraction and identification, the diatom cells were harvested from both LA and LW cultures during the exponential phase. For NMR metabolites analysis, three replicates were used while four replicates were used for GC–MS analysis following [34] procedures. The harvested cells were collected in 50 ml falcon tubes and centrifuged by Avanti J-26 XP Centrifuge Beckman Coulter for 20 min (minutes) at 3700 rpm and at 4°C. The wet pellets were then transferred to small cryovials; washed three times using Milli-Q water to get rid of any salted remaining medium and the pellets were stored at −80°C until extraction. At the time of metabolites extraction, each pallet treated with the solvents listed below to extract the metabolites from the polar (water soluble metabolites) and the non-polar (lipid). All solvents were high performance liquid chromatography grade (HPLC 99.9% purity) and placed in ice prior to the extraction and the extraction solution volume ratio was 2 methanol (MeOH): 1 chloroform (CHCl_3_): 0.8 water (de-ionized H_2_O). At the time of the extraction, the cell pellets were immediately swashed with ice-cold 400 μl MeOH and 125 μl de-ionized H_2_O. The solution was then transferred into a glass vial, vortexed for 30 s (seconds), adding 200 μl CHCl_3_, vortex again for 20s. The solution was then shacked at 350 rpm for 10 min, following an addition of 200 μl CHCl_3_ and 200 μl de-ionized H_2_O, then vortex for 30s. The mixture solution was then centrifuged to clarify the extracts and separate the metabolites layers, where the sample should be biphasic, with a protein debris separating upper (polar) and lower layers (non-polar layer). Using glass Pasteur pipettes carefully each layer was transferred into separate 2 ml Eppendorf cryovials, dried overnight in a speed vacuum and placed in −80°C until later analysis by the NMR. Samples for GC–MS analysis were frozen without drying.

### Nuclear magnetic resonance-based metabolites profiling

After drying, each polar layer sample was dissolved in 500 μl heavy D_2_O water with 0.05% trimethylsilylpropanoic acid (TSP) as an internal reference standard. The non-polar samples were dissolved in 500 μl deuterated-chloroform, vortex plus the TSP for 60 s, then every polar and non-polar sample was transferred directly to a 5 mm NMR tube for acquiring the samples in the NMR. All NMR experiments were performed on a Bruker spectrometer operating at 800 MHz proton frequency. For polar samples, excitation sculpting was used for suppressing of the water peak. For higher NMR data quality, each spectrum was processed in the Topspin software version 4.2.0, the processing included chemical shift referencing, phasing, and baseline correction. Data processing is critical for NMR statistical analysis including the selecting the “interesting spectral regions (alignment and binning) as well as spectral normalization, scaling and transformation” [35].

### Chromatography-mass spectrometry–based metabolites profiling

For GC–MS metabolites profiling, derivatization of metabolites was performed prior to samples preparation. Separately, the derivatization agent mixture was prepared by adding the 10-ppm hydrocarbon mixture (C7-C40, MOX agent) into the BSTFA solution [N,O-Bis(trimethylsilyl)trifluoroacetamide], and incubated in the shaker for 30 min at 37°C at 1500 rpm speed. Calibration curve was prepared by using standards of six different concentrations of amino acids. Method control samples were also prepared as empty cryovials. Pool samples were prepared by mixing all the experimental samples together and used as a quality control samples. In each cryovial (samples, standards and method, and quality control), 50 μl of the derivatization mixture was added, then all cryovials were incubated for 1.5 h at 30°C and 1500 rpm speed in the shaker. For GC–MS analysis, 30 μl of each sample was transferred into the GC glass vials for acquiring the samples.

### Bacterial deoxyribonucleic acid extraction and 16S ribosomal ribonucleic acid gene library preparation

We analysed the bacterial community composition of samples from the three replicated LA and LW treatments as well as samples from the 8-day reciprocal transplant experiment by high-throughput sequencing of the 16S ribosomal ribonucleic acid (rRNA) gene. For each replicate, 200 ml phytoplankton cultures with bacteria were sampled at the end of the algae’s exponential growth. We collected free bacteria samples by performing sequential size-fractionated filtration (3- and 0.2-μm polycarbonate filters) with syringes. DNA extraction was performed with 0.2-μm polycarbonate filters using a PowerWater DNA Kit (MoBio Laboratories, Inc., Carlsbad, CA, USA). The V3 and V4 regions of the 16S rRNA gene were amplified using the primers described by Klindworth *et al.* [36]. Specifically, we used 341F (5′-TCGTCGGCAGCGTCAGATGTGTATAAGAGACAG-CCTACGGGNGGCWGCAG-3′) and 785R (5′-GTCTCGTGGGCTCGGAGATGTGTATAAGAGACAG-GACTACHVGGGTATCTAATCC-3′) primers, both of which contain Illumina overhang adapter sequences at the beginning of the primer sequence.

Two replicates were run in separate polymerase chain reaction (PCR) per sample (final volume 25 μl) using Qiagen multiplex PCR master mix (QIAGEN, Valencia, CA), 2.5 μl of genomic DNA as a DNA template, and a final primer concentration of 0.3 μm. We used the following PCR conditions: initial denaturation at 95°C for 15 min, followed by 30 cycles consisting of denaturation (95°C for 40 s), annealing (55°C for 30 s), and extension (72°C for 30 s), and a final extension step at 72°C for 5 min. Duplicate PCR products were pooled and analyzed by gel electrophoresis (1.5% agarose). The 16S rRNA gene library was prepared following the Illumina 16S metagenomic sequencing library preparation guide. Briefly, the amplicons were cleaned by AMPure XP magnetic bead–based purification (Beckman Coulter, Brea, CA, USA), and MiSeq indices were added via PCR. Indexed PCR amplicons were cleaned by AMPure XP magnetic bead–based purification, quantified using a Qubit 2.0 Fluorometer (Invitrogen, Carlsbad, CA, USA), and pooled equimolarly. A KAPA SYBR FAST Universal qPCR kit with Illumina Primer Premix (Kapa Biosystems Ltd., London, UK) was used for pool quantification, after which pool size was evaluated using a Bioanalyzer (Agilent Technologies, Santa Clara, USA). Then, 6 pM of the pool was sequenced on the Illumina MiSeq platform with 25% PhiX control at KAUST Bioscience core lab facilities. The 16S libraries were sequenced using 2 × 300 bp paired-end reads and a MiSeq reagent kit v3 (Illumina, Inc.).

### Transplant experiment

The cultures that had undergone ~2 years of selection under ambient (LA) and warming conditions (LW) were exposed to the reciprocal ambient (26°C) or warming (30°C) temperature (i.e. reciprocally transplanted) and allowed to grow for 8 days in triplicate 200 ml flasks. Other culture conditions (e.g. medium, light intensity) remained identical to those used in the long-term selection experiment. Bacterial and *C. tenuissimus* cell abundance was monitored daily using methods described above. At the end of the experimental time, the microbiome community was analysed by bacterial DNA extraction and 16S rRNA gen amplification as described above.

### Sequence analysis

Primer sequences were removed using cutadapt [37], after which the divisive amplicon denoising algorithm 2 (DADA2) pipeline (dada2 package version 1.16 in RStudio) was used to analyze the sequencing data. The DADA2 microbiome pipeline (available at https://github.com/benjjneb/dada2) describes microbial communities using unique sequence variants, known as amplicon sequence variants (ASVs) [38]. All functions were run using the recommended and default parameters (https://benjjneb.github.io/dada2/tutorial.html). Taxonomic classification of the selected ASVs was performed with the Silva reference database (Silva reference files, Release 138). Singletons and reads belonging to mitochondria, chloroplasts, and eukaryotes were excluded from further analyses. Species-level identifications were made in the case of a 100% BLAST match; otherwise, identifications were made at the genus level. Unique strains within the same genus were differentiated by adding a unique lowercase letter. All sequenced reads were deposited in the ENA database under the project accession number PRJEB56151.

### Statistical analyses

The final ASV table was divided by treatment, after which we evaluated the distribution of bacterial ASV relative abundance in each treatment within different taxonomic levels (order, family, and genus). Downstream analyses were performed in RStudio (version 1.4.1717) using the phyloseq package and results were visualized using the ggplot2 package in the statistical software R (R Core Team, version 3.6.1). Metabolanalyst software was used for statistically analyzing the peaks from the samples. Chenomx NMR Suite software version 9.0 was used for metabolites profiling and concentrations. For GC–MS based metabolites profiling the Compound Discoverer software version 3.3.2.31 was used for statistical analysis and metabolites profiling. We estimated alpha diversity using the Shannon diversity index, species evenness, and richness. Canonical analysis of principal coordinates (CAP) was used to test whether bacterial community changes were associated with strain selection or assay temperature using the Bray–Curtis distance between samples. We tested the significance of the resulting ordination with a permutational multivariate analysis of variance (PerMANOVA) test (*adonis* function from the vegan package in R) with 999 permutations. A mixed-effects model was used to examine the interactions between strain long-term evolution and assay temperature conditions. For this analysis, the response (e.g. growth rate) was considered the dependent variable, while evolved conditions and assay conditions acted as the fixed effects, and the replicate was treated as a random effect nested within treatment. Differences among sample types were tested using a *t*-test and comparison of means by a student’s *t*-test, and interactions between strain association and temperature were tested by a two-way ANOVA, using JMP 13.0 (SAS) software.

## Results

### Growth responses

**Figure 1:**
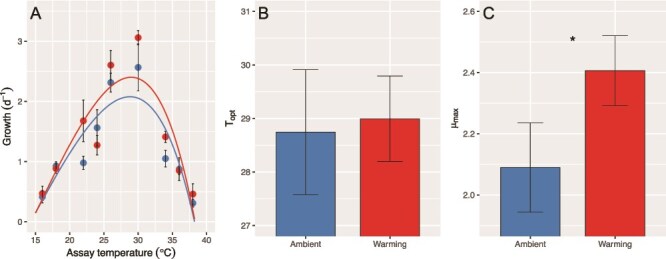
Microbiome thermal performance after long-term adaptation. Patterns of thermal responses of *C. tenuissimus* associated microbiome adapted to LA (26°C; blue) and warming (30°C; red) temperatures. A thermal reaction norms for bacterial growth rates. Solid lines show the thermal reaction norms based on the thermal model. b optimal temperature for growth (*T*_opt_) and c maximum bacterial growth rates (μ_max_) at ambient (blue) and warming (red) temperatures, where columns represent means and bars the standard error (*n* = 3). Asterisks indicate significant differences between ambient and warming treatments based on a student’s *t*-test (*P* < .05).

The microbiota associated with *C. tenuissimus* did not show significant changes in *T*_opt_, corresponding to 28.7 ± 1.2°C and 29.0 ± 0.8°C for LA and LW, respectively (*t*-test, *t* = 0.307, *P* = .774) (Fig. 1). The LW bacteria showed significantly higher μ_max_ than LA groups (LW: 2.41 ± 0.11, LA: 2.09 ± 0.15; *t*-test, *t* = 2.961, *P* = .042; Table 1). The critical thermal minimum (*CT_min_*) and critical thermal maximum (*CT_max_*) did not shift after LW with respect to LA bacterial populations (Table 1). No significant differences in thermal breadth (expressed as *B_80_*, in °C) between LA and LW bacteria populations were detected (Table 1).

**Table 1:** The maximum growth rate (μ_max_), optimal temperature for growth (*T*_opt_, ^o^C), critical thermal minimum (*CT_min_*, ^o^C), critical thermal maximum (*CT_max_*, ^o^C) and 80% performance breadth (*B_80_*, ^o^C) of *C. tenuissimus* and its bacterial microbiome after LA and LW conditions. The thermal reaction traits were derived from the nonlinear curve fitting according to Equation (4) based on their thermal reaction norms (Fig. 1). Data are presented as means ± SE. The superscripts letters represent the significant difference (*P* < .05) between ambient and warming treatments based on a student’s *t*-test. The traits that do not share the same letter had significantly different means. *C. tenuissimus* data from reference [5].

		μ_max_	*T*_opt_	*CT_min_*	*CT_max_*	*B_80_*
*C. tenuissimus*	Ambient	2.41 ± 0.08^a^	29.9 ± 0.16^a^	10.4 ± 1.06^a^	41.31 ± 0.10^a^	12.66 ± 0.16^a^
	Warming	2.50 ± 0.07^b^	31.1 ± 0.10^b^	13.4 ± 0.99^a^	41.66 ± 0.12^b^	11.71 ± 0.35^b^
Bacteria	Ambient	2.09 ± 0.15^a^	28.74 ± 1.17^a^	14.2 ± 1.06^a^	38.19 ± 0.30^a^	10.10 ± 0.60^a^
	Warming	2.41 ± 0.11^b^	28.95 ± 0.80^a^	14.2 ± 0.99^a^	38.39 ± 0.23^a^	10.00 ± 0.35^a^

We found a close relationship between bacterial and diatom growth (Fig. S1). Bacterial growth followed that of phytoplankton after a lag phase of 3 days when evolving under LA and 4 days under LW (Fig. S1)—a pattern consistently observed among the replicates. However, when the algae reached the stationary phase, the maximum number of bacterial cells in LW microbiome was significantly lower than that of LA (Fig. S1), and this difference remained when communities were transplanted to reciprocal temperatures (strain selection × assay temperature interaction, two-way ANOVA, *F*_1,8_ = 343.174, *P* < .001). When LW and LA communities were exposed to reciprocal temperatures, bacterial growth in the LW community continued to be significantly higher than that of LA populations during the exponential growth examined, regardless of the change in assay temperatures (strain selection × assay temperature interaction, two-way ANOVA, *F*_1,8_ = 7.207, *P* = .028) (Supplementary Fig. S1).

### Nuclear magnetic resonance–based metabolome

**Figure 2:**
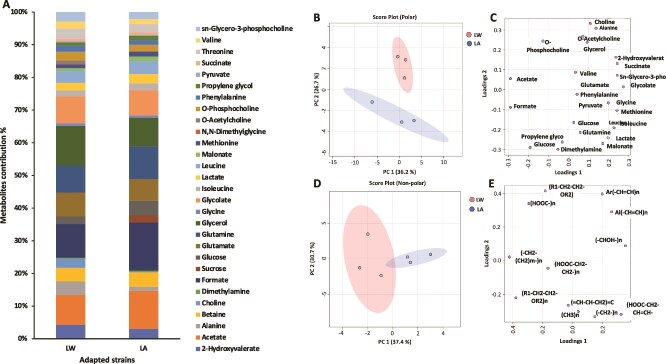
^1^H NMR-spectroscopy metabolites. (A) Percentage contribution of the fraction assignments of metabolite abundance to the total metabolites concentration obtained in both LW and LA temperatures *C. tenuissimus* adapted strains. B and D (score plots) and D and E (loading plots) principal components analysis of non-polar (B and D) and polar (D and E) metabolites from long-term warm (LW, red dots) and LA (blue dots) temperature adapted strains of *C. tenuissimus*.

We successfully assigned each peak for the identified metabolites from the polar layer of each LW and LA *C. tenuissimus* adapted strains (Supplementary Fig. S2) by combining ^1^H NMR spectroscopy and Chenomx metabolites identification library (Chenomx Compound Library). We detected water soluble primary metabolites ranging from 28–38, and ~10 unknown metabolites in the regions of 1.23, 1.28, 2.02, 2.12, 2.23, 2.73, 2.8, 3.93, and 3.95, 4.8 ppm (Supplementary Fig. S2). The NMR metabolomic profiles of polar (water soluble metabolites) showed the presence of the key primary metabolites such as amino acids (alanine, valine and leucine, etc.), organic compounds (formate, succinate, acetate, etc.), and carbohydrates mainly glucose (Table S1). However, primary compound concentrations were generally low, with some, like sugars in the LW strain, falling below detection limits (Fig. 2A; Table S1). Overall, LW samples had higher amino acid concentrations (mean ± SE, 46.53 ± 0.55%) compared to ambient conditions (39.46 ± 0.63%, Fig. 2A). Carbohydrates were significantly higher in LA samples than in LW samples (One-way ANOVA, F = 9.06, df = 1, *P* = 0.03), but contributed minimally to total metabolites in both strains: 6.65 ± 0.01% (LA) and 2.37 ± 0.01% (LW) (Fig. 2A). Other organic compounds showed similar contributions in both strains, averaging 53.89 ± 0.02% (LA) and 51.10 ± 0.01% (LW).

**Figure 3:**
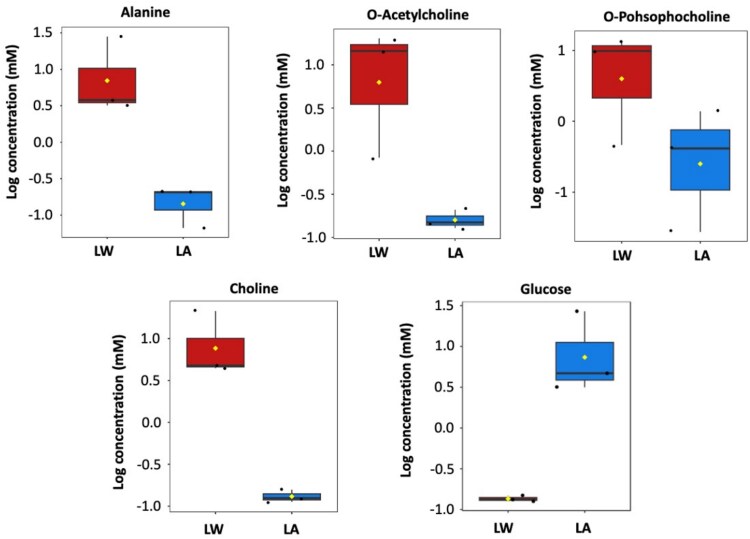
^1^H NMR-spectroscopy polar metabolites. Concentrated metabolites presented in adapted strains to long-term warm (LW, red box) and LA (blue box) temperature, respectively. The black dots represent the concentrations from the replicated samples, the notch indicates the 95% confidence around the median of each group. The mean concentration of each group is indicated with a yellow diamond. All differences were significant ^*^P < .01.

The spectra from each treatment group, both polar and non-polar (Fig. 2 B,C), aligned well within the first two principal components. PC1 captured 36.2 % (LA) and 37.4 % (LW) of the variance, separating the samples into two categories. The loading plots for PC1 and PC2 across LW and LA for both polar and non-polar revealed (Fig. 2 D,E) that non-polar metabolites showed no significant differences between the two LW and LA adapted strains. However, for polar metabolites, amino acids such as alanine, choline, o-phosphocholine, and o-acetylcholine were more abundant in the LW strain, while carbohydrates like glucose were higher in the ambient-adapted LA strain (Fig. 3). To quantitatively explore the variations of the non-polar fractions (Table S3), we compared the prevalence of different compounds between LW and LA adapted strains. We added up the total area of these assignments for each strain and then calculated the percentage of each assignment. (Supplementary Fig. S3). Although there were slight differences in the combined percentages of each assignment between the two groups (as shown in Table S3), statistical analysis revealed no significant distinctions. However, it was noticeable that compounds like (-CH_2_-(CH_2_)m-)n, which represent varying triglycerides, lipids, and cholesterol methyl groups, along with (Y-CH_2_-CH_2_-X) found in sterols, and (HOOC-)n in fatty acids, began to accumulate in the LW *C. tenuissimus* adapted strain.

### Gas chromatography-mass spectrometry–based metabolome analysis

**Figure 4:**
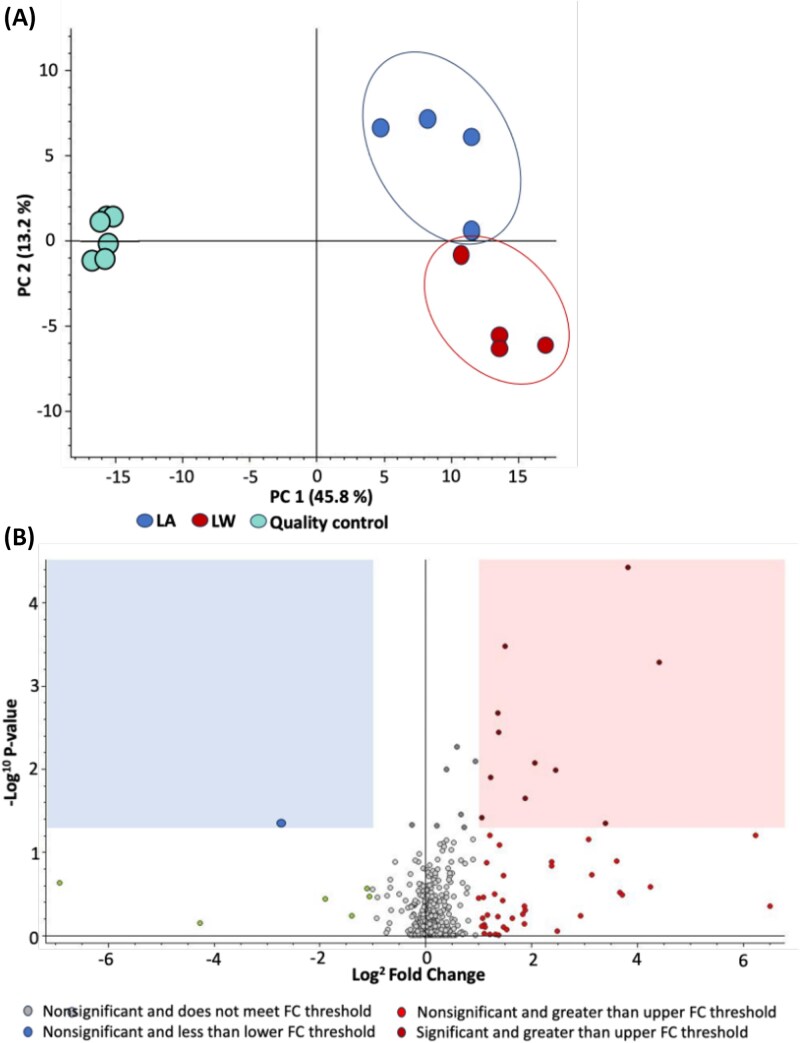
GC–MS metabolome analysis. (A) Principal component analysis (PCA) score plot of the LW, LA and the pool samples from both strains (quality control). (B) Scatter plot of the assigned metabolites that contributed the most to the clustering in the PCA analysis.

Since NMR-based metabolome analysis are less sensitive than MS based analysis, we combined the NMR data analysis with the data from GC–MS for better examination of strain’s differences. We successfully detected a total of 258 metabolites including unknown peaks, 126 known metabolites, and 61 derivatives metabolites including amino acids, organic acids, and sugars. Water soluble metabolic features was clustered depending on the diatoms adapted strain condition LW and LA in addition to the pool samples (quality control) as indicated by PCA in Fig. 4A. The scatter plot showed some featured metabolites including unknown peaks contributing to the separation among the two LW and LA clusters (Fig. 4B).

Among the metabolites contributing to the differences between adapted strains, several derivatized carbohydrates were significantly more concentrated in LA including, but not limited to glucose, 8TMS; retention time (RT) 23.054 (Fig. S4A), Maltose isomer 1, 1MOX, 8TMS; RT 23.325 & 23.718 (Fig. S4B), Isomaltose isomer 2, 1MOX, 8TMS; RT 23.416 & 23.568 (Fig. S4C). In contrast, glutamic acid levels were elevated in LW strain (Fig. S4D). A large proportion of the unknown peaks were more concentrated in LW, along with several amino acids and organic compounds. To further investigate metabolite changes between the two adapted strains, we conducted a fold change analysis (Table S3). The data showed that, compared to LA, LW exhibited a higher accumulation of amino acid metabolites and a lower accumulation of carbohydrates, suggesting a metabolic shift in response to adaptation to LW conditions (Table S3).

### Partnerships changes

**Figure 5:**
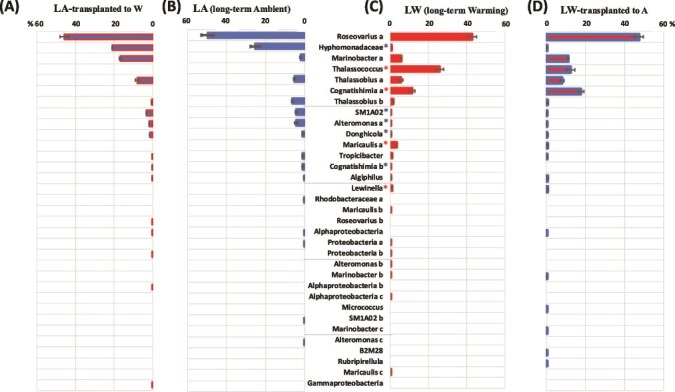
Partnerships strength. Changes observed in *C. tenuissimus* microbiome community. Columns represent the mean percent relative abundance of ASVs reads identified to the genus level after (A) LA and (B) LW selection. Changes in the bacterial microbiome after the short-term reciprocal transplant experiment are provided where (A) represent LA was transplanted to 30°C (blue fill with red border), (B) LA (26°C; blue), (C) LW (30°C; red) and (D) LW transplanted to 26°C (red fill with blue border). Blue and red asterisks indicate significantly higher percent relative abundances (student’s *t*-test, *P* < .0001) in the ambient and warming strains, respectively. Letters after the genus name indicate different ASVs.

The microbiome associated with the LA and LW *C. tenuissimus* strains were dominated by the phylum Proteobacteria, which represented >95% of the total reads (Supplementary Fig. S4). At the order level, the Rhodobacterales (consisting of the family Rhodobacteraceae) dominated the communities regardless of selection temperature (Fig. S4) but showed significantly higher abundance in the LW community (87.6 ± 0.8%; mean ± SE) than in the LA group (60.2 ± 2.2%) (*t*-test, Table S4, *P* < .0001). The abundance of Caulobacterales, however, significantly increased in the bacterial communities associated with LA strains (23.3 ± 0.7%) relative to that of LW strains (2.5 ± 1.4%) (Table S4, *P* < .0001) (Fig. S2). While a small proportion of Phycisphaeraceae (3.76 ± 0.29%) was detected in the LA populations, this family was greatly reduced in the LW populations (0.01 ± 0.009%) (Fig. 5). One member of the genus *Roseovarius* (identified as *Roseovarius* sp*.*) dominated the community of bacteria associated with both LA (49.6 ± 2.9%) and LW (42.8 ± 2.4%) strains (Fig. 5). There were several genera common to the two long-term selection strains (Fig. 5), although some genera strains (*Thalassococcus*, *Cognatishimia-a*, *Maricaulis-a*, *Lewinella*, and *Marinobacter-b*; Fig. 2) appeared much more abundant in communities associated with the LW strain; specifically, a *Thalassococcus* sp*.* and *Cognatishimia-a* sp. made up a considerable proportion of the LW community, with 25.7 ± 2.1% and 11.5 ± 1.45% of reads, respectively (Fig. 5C). These genera remained present after brief exposure to ambient temperatures in the transplant experiment (Fig. 3D). Other bacterial genera associated with the LA diatom strain included *Hyphomonadacea* (Fig. 5A and B), which represented >20% of reads, but had low abundance in the LW community (0.8% of reads; Table S4, *P* < .0001). The short-term exposure of the LA community to warming shaped bacterial community structure by reducing the number of taxa (Fig. 5A and B; Table 2) but significantly increasing the abundance of *Marinobacter-a* (from ~2% to ~17%) (Table S4, *P <* .0001).

**Table 2:** Diversity changes after coevolution. Mean (±SE) values of species richness, Shannon diversity index, and evenness (EVAR) of the bacterial microbiomes associated with *Chaetoceros tenuissimus* strains after long-term adaptation to ambient (LA) and warming (LW) temperatures, and when exposed to the reciprocal temperature (in italics). Significant differences were observed for richness (*P* = .026). Values labeled with the same letter (a, b) are not significantly different (*P* > .05, *t*-test comparison of means). SAV’s extinctions are relative to the LA community.

	Ambient strain (LA)		Warming strain (LW)		
	Long-term A	Short-term transplanted to warming	Long-term W	Short-term transplanted to ambient	
Richness	13.33^b^	13.33^b^	17.00^a^	15.67^ab^	
	± 0.88	± 0.88	± 1.15	± 0.88	
Shannon	1.51	1.52	1.64	1.54	
	± 0.04	± 0.03	± 0.06	± 0.05	
EVAR	0.11	0.11	0.09	0.09	
	± 0.02	± 0.01	± 0.01	± 0.01	
Extinctions	-	4	6	5	

**Figure 6:**
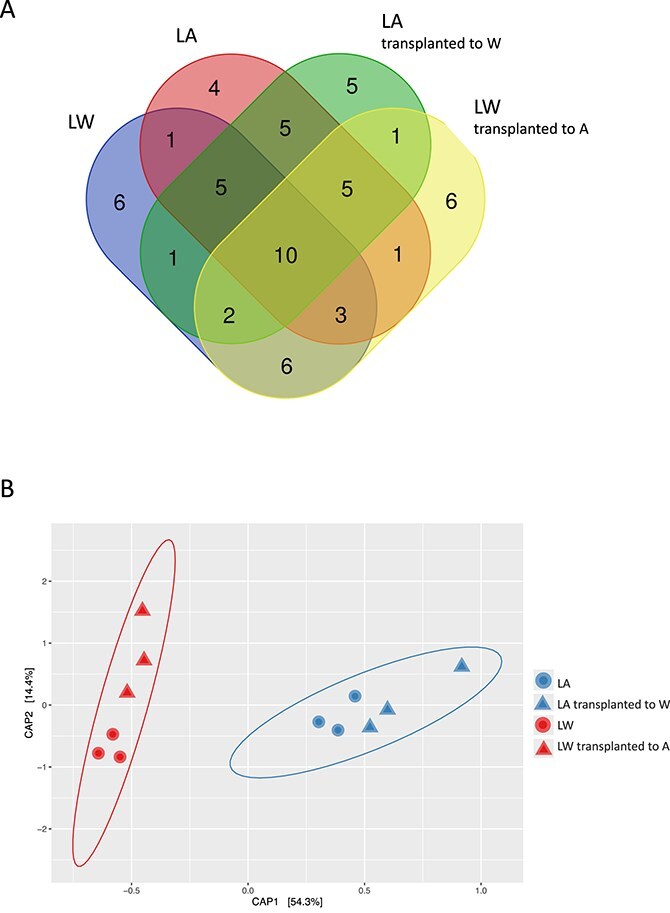
Beta diversity. (A) The overlapping relationships of *C. tenuissimus* microbiome communities associated to the two long-term and transplanted strains, indicating the number of similar SAVs. (B) CAP based on *C. tenuissimus* associated bacterial ASVs microbiome after long-term temperature adaptation. Blue symbols correspond to LA temperatures (26°C; circles) and red symbols denote LW conditions (30°C; circles). Triangles correspond to the *C. tenuissimus* microbiome after transplant to reciprocal temperature conditions. Each point represents a replicated sample. CAP was calculated using Bray–Curtis distances with a multivariate *t*-distribution.

The total number of ASVs was highest in the LW microbiome, which had a maximum of 22 and a mean of 17 ASVs, while the LA community had a mean of just 13.3 ASVs (Table 3); the two groups shared a mean of 10 ASVs (Fig. 6A). When the LA populations were transplanted to warming temperatures, they experienced no change in richness, but when LW populations were transplanted to ambient temperatures, there was a small reduction in richness to 15.7 ASVs, and shared a larger number of ASVs with LW than with LA (Fig. 6A). Diversity (Shannon index) and evenness were overall low in the bacterial communities, and although we observed some increase in diversity in the LW group, the differences between LW and LA community diversity were not significant. Similarly, though evenness was slightly higher in the LA community, differences in mean evenness were not significant (Table 2). Extinctions, calculated as the disappearance below the detection of ASV reads relative to the LA community, were greater in the LW group, which was compensated with the appearance of new partners (Table 2).

In addition, we found that the *C. tenuissimus* microbiome communities differed significantly after long-term temperature treatments (PerMANOVA, *P* = .002). CAP indicated that there were substantial changes to community structure associated with the unique, evolved strains that explained the most variance in the model (54.3%) (Fig. 6B). After the reciprocal transplant experiment, the bacterial community generally remained similar to that associated with the selected strains, and exposure to reciprocal temperatures explained a smaller variance of 14.4% (Fig. 6B).

## Discussion

Our experiment evaluated the coevolution of a diatom (*C. tenuissimus*) and its bacterial microbiome after long-term temperature selection pressure. Warming adaptation altered the diatom’s thermal response, increasing amino acids and decreasing carbohydrate storage. The microbiota raised μmax without shifting T_opt,_ while one genus dominated throughout. Warming also introduced new partners, increasing species richness and reshaping the core microbiome.

A previous study showed that *C. tenuissimus* adapted to warming by shifting its T_opt_ and increasing its μ_max_ [5]. The increased growth rate in LW led to a higher number of generations, potentially accelerating the adaptation process. After ~2 years of warming selection, we observed that the bacterial microbiome also increased its μ_max_ without altering T_opt_ or thermal limits (CT_min_, CT_max_). This parallel increase in growth rates supports findings that mutualisms often drive trait complementarity and matching between partners [39]. However, the microbiome evolving under warming showed lower maximum abundance when the algae reached the stationary phase and a 1-day lag in growth, possibly due to community adjustments. Warming led to reduced *Caulobacterales* abundance and the introduction of *Thalassococcus*, *Cognatissima*, *Lewinella*, and *Marinobacter*, absent under ambient conditions. Recent studies suggest bacteria–diatom and bacteria–bacteria interactions select for simplified associations, adaptable over long-term cultivation [40], consistent with the low diversity seen in our microcosms. Moreover, the core bacterial community composition was more influenced by long-term adaptation than by short temperature changes.

Diatoms often associate with specific bacterial taxa, mainly phyla Proteobacteria and Bacteroidota [27]. Our study confirmed this, with Proteobacteria constituting more than 95% of the *C. tenuissimus* microbiome. Planctomycetes were the second-most abundant phylum, but were replaced by Bacteroidota when the communities were transplanted to the reciprocal temperature. The dominant order, Rhodobacteriales, are one of the major groups of marine bacteria, comprising up to 20% of coastal and 15% of mixed-layer ocean bacterioplankton communities [41] and references therein. This order is commonly associated with algae [42, 43] and diatoms [27, 44] and the Rhodobacteriaceae family, considered to have many “ecological generalists” and known for its ecological versatility, dominated the microbiome [41, 45, 46]. Similarly, the abundance of the order Alteromonadales increased significantly when both the LW and ambient communities were exposed to reciprocal temperatures. These bacteria are a widely distributed group of Gammaproteobacteria [47] but are believed to be sparsely present in marine environments [42]. Transcriptomics analysis of these heterotrophic bacteria revealed that Alteromonadaceae includes many “ecological specialists” that grow rapidly, expressing metabolism genes related to nitrogen assimilation, fatty acid catabolism, and the TCA cycle [48]. Although short-term temperature stress led to a significant increase in their abundance, we assume that differences in the substrates produced by the algae may also influence [49]. *Roseovarius* sp., the dominant bacterium in the *C. tenuissimus* microbiome, is commonly found in Red Sea water and sediment bacterial communities and is also part of the microbiomes of other organisms, such as corals [50, 51]. Certain *Roseovarius* strains are known to aid in stress tolerance and have been used to inoculate in symbiotic corals to enhance their thermal resilience [52].

Planktonic microalgae and bacteria coexist at the ocean’s surface and engage in complex interactions that influence biogeochemical cycles [27]. Their interactions can lead to mutualism, parasitism, predation, or competition. While bacterial parasitism is common [53], and competition for nutrients like phosphate occurs in nutrient-deficient systems [54], most interactions are beneficial. Phytoplankton provide dissolved organic carbon (DOC) to bacteria, shaping bacterial diversity [55]. In return, bacteria offer phytoplankton vitamins [56] or siderophores that bind iron [11]. Thus, mutualism is the most frequent and significant interaction, and is an important process impacting ocean biogeochemistry [29, 57].

Our comparative analysis of the NMR and GC–MS-based metabolomes revealed both complementary and overlapping insights into the metabolic profiles of LW and LA strains. While GC–MS is known for its higher sensitivity and ability to detect a broader range of metabolites, particularly volatile and derivatized compounds, NMR provides quantitative and structural information on metabolites in their native state [58]. Our findings from the NMR and GC–MS metabolomics-based analysis revealed that amino acids were more accumulated in LW strain than in LA, while non polar lipids content showed no significant changes. Using both techniques, we detected over 296 metabolites from different classes of amino acids, carbohydrates and organic molecules. A substantial number of unknown peaks (136) could not be identified using the reference libraries Discoverer Compound and Chenomx software, highlighting a significant limitation in current marine algal metabolomics. Some of these unknown metabolites contributed to the differences between the LW and LA adapted strains, emphasizing the need to expand marine-specific metabolite databases and develop advanced tools, such as machine learning for compound annotation and high-resolution structural analyses. Despite this limitation, our study detected more metabolites than previous metabolomics studies on diatoms such as *Chaetoceros tenuissimus* [59]. While GC–MS identified a broader range of volatile and derivatized compounds, NMR provided quantitative and structural information in native conditions [34]. Ten out of the 38 metabolites detected via NMR were also detected by GC–MS, mostly amino acids involved in the TCA cycle and protein biosynthesis demonstrating the complementary nature of both platforms. Both strains shared 90% of the identified metabolites, suggesting that concentration differences, rather than profiling, drove the observed separation. Amino acids and sugars clearly differentiated the two strains. Choline was uniquely abundant in the LW strain via NMR but undetectable by GC–MS (Fig. S5), likely due to differences in compound volatility and derivatization efficiency. As a precursor to membrane phospholipids, increased choline may indicate alterations in membrane composition, to maintain fluidity and integrity under temperature stress [60].

Notably, glucose was nearly undetectable in the LW strain, but highly expressed in the LA strain via GC–MS. Conversely, choline was abundant in the LW strain but undetected in the LA strain. This likely reflects increased glucose mobilization in LW to meet energy demands under warming stress, as rapidly growing cells often consume glucose immediately [61].

Diatoms exhibit metabolic plasticity in response to environmental stressors, adjusting key biochemical pathways to optimize survival and function [6]. In general, amino acids were significantly elevated in the LW-adapted strain—up to five-fold for certain compound—contributing ~47% of total metabolites in the NMR dataset. These molecules are central to protein synthesis and are tightly linked to central carbon, nitrogen, and sulfur metabolism [61]. Their accumulation aligns with adaptive response to temperature stress, maintaining biosynthesis capacity and metabolic flexibility [62]. Some amino acids, such as L-glutamic acid and L-phenylalanine, increased significantly in LW (5.21-fold and 3.17-fold, respectively), whereas others, like leucine and threonine, decreased. This suggests not simple accumulation but a dynamic metabolic flux, with selective synthesis, utilization, or degradation in response to physiological needs [62–64]. Elevated glutamic acid may also reflect improved nitrogen assimilation [61]. Interestingly, despite previous studies linking warming with increased saturated fatty acid accumulation [65, 66], our data showed no significant changes in non-polar lipids between the two strains. This highlights amino acid metabolism as the primary biochemical shift in the LW strain and a likely key to thermal adaptation.

Diatom metabolite changes can profoundly influence their interactions with microbiomes [27]. Our findings on the downregulation of sugar metabolites under warming align with findings in *Skeletonema dohrnii* and *Roseobacter* sp., while upregulated amino acids, such as alanine, promoted bacterial nucleotide synthesis [67]. Our metabolomics approach yielded valuable insights into the metabolic products released by the host and contributed to understanding microbiome community responses. However, integrating metatranscriptomics (to capture functional activity), metagenomics (for a deeper taxonomic profiling) to metabolomics would provide a more comprehensive view of microbiome dynamics and their functional shifts [68]. Our results showed that warming adaptation altered both metabolite diversity and composition. Adaptation to warming lead to more extinctions among low-abundant bacterial ASVs, indicating their higher sensitivity to temperature shifts compared to dominant genera. Previous studies suggest that a few “super-generalist” species play a key role in shaping evolution and coevolution in diverse communities [10, 69, 70]. Generalized mutualisms may buffer environmental disturbances by favoring resistant species [71]. However, niche specialization may exist within seemingly redundant mutualisms, where partners offer complementary benefits [72]. This complexity emphasizes the fragility of interactions under environmental change. Over time, small differences in partner quality can have significant evolutionary impacts.

Adaptation will be necessary to avoid the sharp decline in tropical phytoplankton diversity predicted in the absence of an evolutionary response to warming [31]. However, thermal adaptation poses a significant challenge for marine organisms, a process further complicated by concurrent environmental stressors associated with global change such as ocean acidification, increased stratification, and pollutant exposure. The cost of thermal adaptation, is likely to alter the organism’s response to other environmental stressors [73]. However, in the case of *C. tenuissimus* from the Red Sea, thermal adaptation conferred a degree of cross-tolerance to heavy metal exposure [74], suggesting that the underlying physiological or metabolic modifications may enhance resilience to other environmental stressors. To fully understand the dynamics between host organisms and their microbiomes in the global changing environment, future research should include the complex interplay between climate warming and other environmental stressors. Moreover, a larger number of species must be included to broader the results observed.

Our study revealed significant shifts in the core bacterial microbiome of *C. tenuissimus* after long-term warming. The observed increase in bacterial microbiome is consistent with findings from plants and animal’s studies, which suggest that asymmetries in co-evolutionary networks can support long-term coexistence, promote biodiversity, facilitate the acquisition of new partners and lead to evolutionary shifts [10, 75]. Our results showed significant changes in algal metabolites due to long-term temperature adaptation. Therefore, our experimental results with marine microbial communities underscore the importance of long-term coevolution in maintaining coexistence and driving shifts in microbiome interactions under warming conditions.

## Supplementary Material

Supplementary_Information_ISMEcommFinal_ycaf103

## Data Availability

Data and code related to this publication can be found in the ENA database under the project accession number PRJEB56151 and PQ480815Genbank accession number.
