# Supplementary material for: Host metabolite production and microbiome dynamics: effects of long-term diatom adaptation to warming
Source: ISME Commun. 2025 Jun 19;5(1):ycaf103. doi: 10.1093/ismeco/ycaf103 (PMC12743300; doi:10.1093/ismeco/ycaf103)
Supplement: Supplementary_Information_ISMEcommFinal_ycaf103 [file supplementary_information_ismecommfinal_ycaf103.docx]

**Supplemental Material**

**Host Metabolite Production and Microbiome Dynamics: Effects of Long-Term Diatom Adaptation to Warming**

**Susana Agusti ^1^, Afrah Alothman^1^, Peng Jin^2^, Clara S. Stanschewski ^1,3^, Rubén Díaz-Rúa^1^, Abdul-Hamid Emwas^4^, Upendra Singh^5^, and Mariusz Jaremko^5^**

**Figure S1:** Cell number changes (expressed as Ln cell number) of associated bacteria (red) and phytoplankton (blue) as a function of incubation days in the reciprocal transplant experiments. a, Long-term ambient (26 ^o^C) selected bacteria assayed at ambient temperature (26 ^o^C); b, Long-term ambient (26 ^o^C) selected bacteria assayed at warming temperature (30 ^o^C); c, Long-term warming (30 ^o^C) selected bacteria assayed at ambient temperature (26 ^o^C); d, Long-term ambient (30 ^o^C) selected bacteria assayed at warming temperature (30 ^o^C). Data are the means ± SD, *n* = 3.

**Figure S2**: S ^1^H proton NMR 800-MHz spectral information and metabolites assignments of (A) polar metabolites assignments of the region between 0.5-3.0 ppm, and (B) the polar metabolites of the regions between 3.0-9.0 ppm of the two treatments long-term ambient (LA, green spectra), and long-term warm (LW, red spectra) adapted strains. X-axis presents the chemical shifts of the entire spectra, whereas the numbers above each peak represent identified metabolite. The polar metabolites were identified and presented for each peak as: 2-Hydroxyvalerate (1); leucine (2); Isoleucine (3); Valine (4); Propylene glycol (5); unknown (6); unkonwn (7); lactate (8); threonine (9); alanine (10); acatate (11); unknown (12); mthionine (13); unkown (15); glutamine (16); glutamate (17); pyruvate, succinate (18); dimethylamine (19); unkown (20); unkown (21); N,N dimethylglycine; trimethylamine (23); betaine (24); choline (25); malonate (26); O-acetylcholine (27); O-phosphocholine (28); sn-glycero-3-phosphocholine (29); glucose (30); unknown (31); unkown (32); sn-glycero-3-phosphocholine (33); unkown (34); glucose (35); glucose (36); phenylalanine (37); unkown (38).

**Figure S3**: **Non-polar compounds-** The contribution of each fraction assigned region to the total metabolites regions in both long-term ambient (LA) temperature and long-term warm temperature (LW).


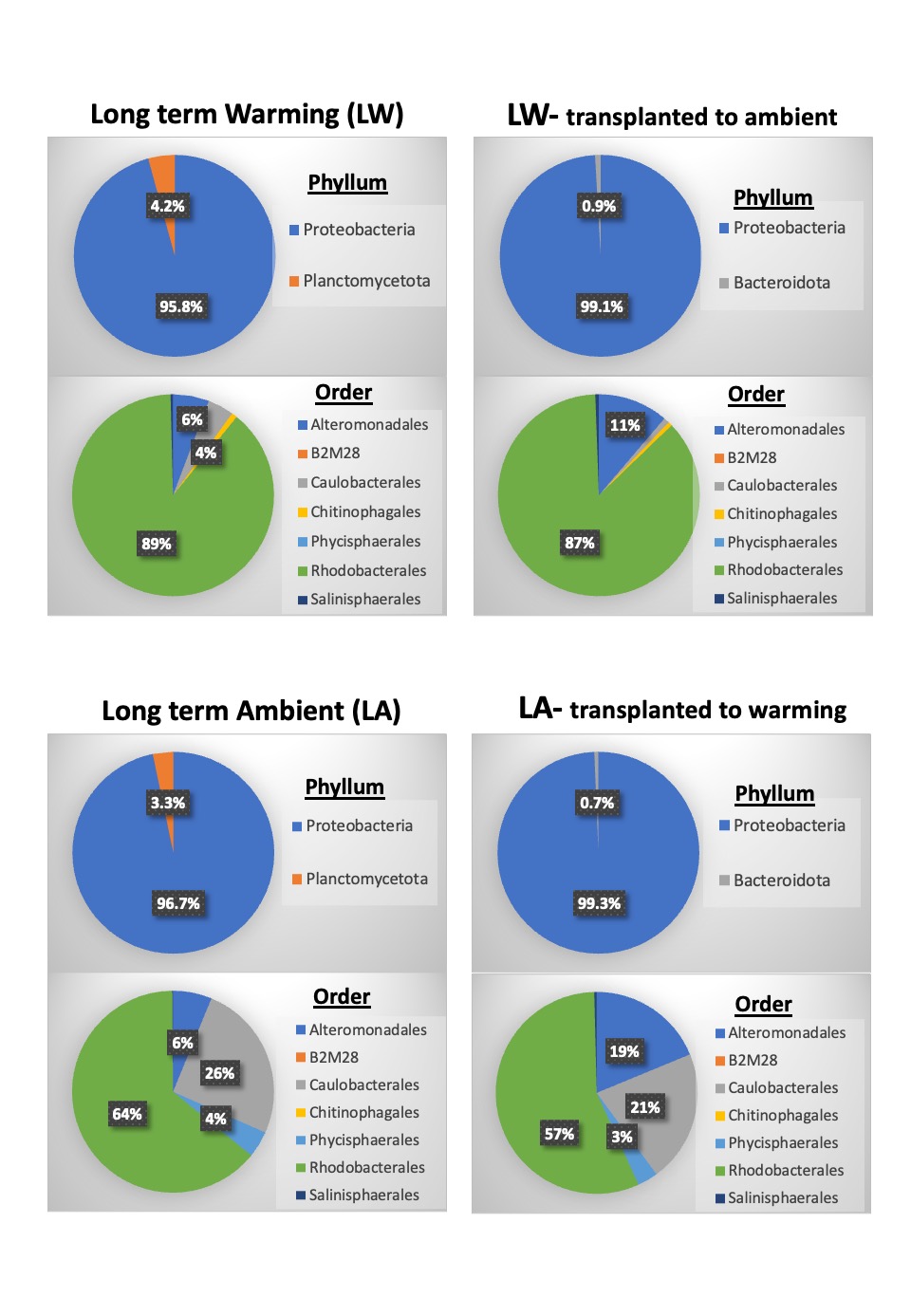
 **Figure S4.** Bacterial community associated to the microalgae *C. tenuissimus* after long term adaptation to warming (LW, 30oC) and ambient (LA, 26oC) temperatures, and when transplanted for short time to reciprocal temperatures.


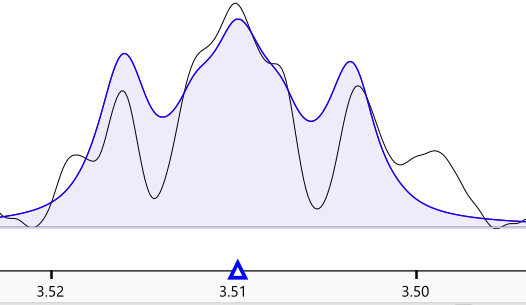

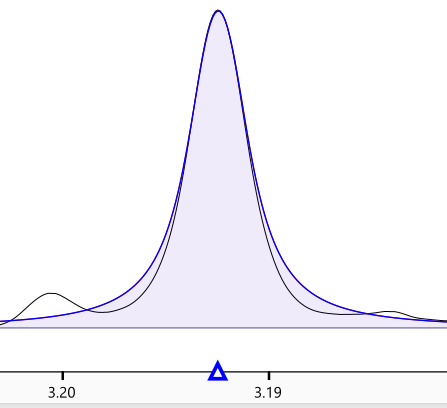

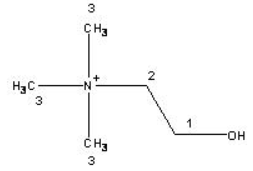

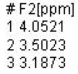

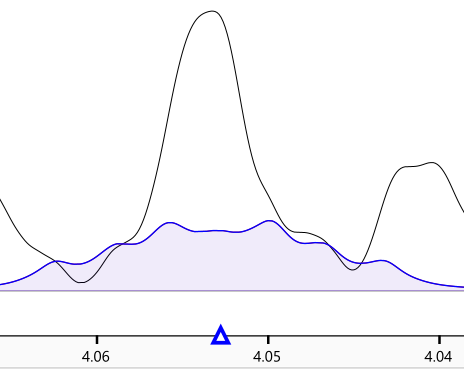


(a)

(b)

(c)

(d)

2

3

1

**Figure S5**. 1D ^1^H NMR spectra show annotated signals of choline metabolite that are systematically annotated by their spectral signals with the standardized reference libraries Chenomx where two multiplets of methylene groups numbered 1 and 2 at chemical shifts 4.05 ppm (a), and 3.52 ppm (b), respectively and a singlet of methyl group number with 3 at 3.19 ppm (c), and its chemical structure with numbered and chemical shifts of the reference library human metabolome database (HMDB).

**Table S1:** Mean concentration and standard error (SE), polar exchanges, chemical shifts, and profiles of selected primary metabolites observed by ^1^H NMR spectroscopy and obtained by Chenomx Compound Library and NMR analysis form log-term warm (LW) and long-term ambient (LA) adapted strains of diatom species *C. tenuissimus*.

| Metabolites | Class | LW (mM) | SE | LA (mM) | SE | Chemical shift (ppm) |
| --- | --- | --- | --- | --- | --- | --- |
| Alanine | Amino acid  Aliphatic amine  Aliphatic amine  Aliphatic amine  Amino acid  Amino acid  Amino acid  Amino acid  Amino acid  Amino acid  Amino acid  Amino acid  Amino acid  Aliphatic amine | 0.0032 | 0.0008 | 0.0011 | 0.0001 | 1.50 |
| Betaine |  | 0.0032 | 0.0007 | 0.0039 | 0.0013 | 3.27, 3.9 |
| Choline |  | 0.0021 | 0.0009 | 0.0000 | 0.0000 | 3.20 |
| Dimethylamine |  | 0.0002 | 0.0000 | 0.0004 | 0.0000 | 2.70 |
| Glutamate |  | 0.0055 | 0.0016 | 0.0061 | 0.0004 | 3.7, 2.3 |
| Glutamine |  | 0.0064 | 0.0014 | 0.0092 | 0.0000 | 2.30 |
| Glycine |  | 0.0007 | 0.0003 | 0.0007 | 0.0001 | 3.55 |
| Threonine |  | 0.0024 | 0.0004 | 0.0024 | 0.0004 | 1.30 |
| Valine |  | 0.0016 | 0.0006 | 0.0015 | 0.0005 | 1.00 |
| Isoleucine |  | 0.0014 | 0.0006 | 0.0018 | 0.0005 | 1.00 |
| Leucine |  | 0.0028 | 0.0009 | 0.0036 | 0.0007 | 0.94 |
| Methionine |  | 0.0009 | 0.0001 | 0.0012 | 0.0003 | 2.12 |
| N,N-Dimethylglycine |  | 0.0002 | 0.0000 | 0.0003 | 0.0000 | 2.90 |
| O-Acetylcholine |  | 0.0007 | 0.0002 | 0.0001 | 0.0000 | 3.20 |
| O-Phosphocholine |  | 0.0020 | 0.0003 | 0.0017 | 0.0003 | 3.20 |
| Phenylalanine |  | 0.0014 | 0.0007 | 0.0013 | 0.0003 | 7.30 |
| Propylene glycol |  | 0.0008 | 0.0001 | 0.0013 | 0.0000 | 1.10 |
| Glucose | Carbohydrates | 0.0000 | 0.0000 | 0.0020 | 0.0007 | 5.50, 3.67, 5.4 |
| Glycolate | Organic compounds | 0.0063 | 0.0004 | 0.0070 | 0.0002 | 3.96 |
| Lactate |  | 0.0017 | 0.0001 | 0.0026 | 0.0003 | 1.30 |
| Malonate |  | 0.0007 | 0.0000 | 0.0012 | 0.0003 | 3.20 |
| Pyruvate |  | 0.0003 | 0.0001 | 0.0003 | 0.0001 | 2.40 |
| Succinate |  | 0.0006 | 0.0001 | 0.0006 | 0.0000 | 2.40 |
| sn-Glycero-3-phosphocholine |  | 0.0022 | 0.0005 | 0.0019 | 0.0003 | 3.2, 4.3 |
| 2-Hydroxyvalerate |  | 0.0033 | 0.0004 | 0.0027 | 0.0006 | 0.96 |
| Acetate |  | 0.0069 | 0.0000 | 0.0113 | 0.0053 | 1.90 |
| Formate |  | 0.0078 | 0.0006 | 0.0142 | 0.0050 | 8.00 |
| Glycerol |  | 0.0091 | 0.0014 | 0.0079 | 0.0000 | 3.6, 3.7 |

**Table S2:** Integrated percentage of each assignment found in non-polar extract spectra from long-term ambient (LA) and long-term warm (LW) adapted strains analyzed using NMR.

| Compunds | LW % | LA % |
| --- | --- | --- |
| (-CH_3_)n | 9.34% | 9.56% |
| (-CH_2_-)n | 8.72% | 9.22% |
| (-CH_2_-(CH_2_)m-)n | 34.39% | 32.81% |
| (-CH_2_-(CH_2_)o-)n | 1.61% | 1.63% |
| (OOC-CH_2_-CH_2_-)n | 0.36% | 0.32% |
| (-OC-CH_2_-CH=CH-)n | 2.59% | 2.55% |
| (-N+(CH_3_)_3_)n | 1.13% | 1.36% |
| (-+N-CH_2_-) | 8.44% | 8.40% |
| (Y-CH_2_-CH_2_-X) | 1.47% | 1.20% |
| (Y-CH_2_-CH_2_-X) | 1.33% | 1.20% |
| (-CHOH-)n | 3.29% | 3.82% |
| Al(-CH=CH-)n | 15.01% | 15.40% |
| Ar(-CH=CH-)n | 11.98% | 12.29% |
| (HOOC-)n | 0.33% | 0.24% |

**Table S3**: Log2-transformed mean fold changes (threshold = 1, -1) in diatom metabolites extracted from two different treatments: long-term warm (LW) and long-term ambient (LA) adapted strain. Positive fold change values indicating more concentrated metabolite in the LW treatment comparing to LA treatment.

| Name | RT  [min] | fold change (log2) |
| --- | --- | --- |
| Maltose isomer 2, 1MOX, 8TMS | 23.718 | -7.41 |
| DL-Aspartic acid, 3TMS | 11.207 | -4.5 |
| Propylene glycol, 2TMS | 11.195 | -4.3 |
| Tromethamine, 4TMS derivative | 11.276 | -4.3 |
| Glucose, 8TMS | 23.054 | -3.65 |
| L-Aspartic acid, N-trimethylsilyl-, 4-methyl ester, 1-trimethylsilyl ester (isomer 1) | 9.68 | -3.18 |
| Leucine, 2TMS | 7.413 | -2.87 |
| L-Methionine, 2TMS | 11.156 | -2.5 |
| (Z)-5-Methoxy-3,5-dimethyl-2-hexenyltrimethylsilane | 16.418 | -2.27 |
| Isomaltose isomer 2, 1MOX, 8TMS | 23.416 | -2.15 |
| L-Lysine, 4TMS | 16.865 | -1.77 |
| Cyclononasiloxane, octadecamethyl- | 26.687 | -1.69 |
| L-Ornithine, 4TMS | 15.5 | -1.66 |
| Tyrosine, 3TMS | 16.886 | -1.57 |
| L-Threonine, 3TMS | 9.306 | -1.45 |
| L-Serine, 3TMS | 8.774 | -1.41 |
| Maltose isomer 1, 1MOX, 8TMS | 23.325 | -0.99 |
| Glycine, 3TMS | 7.982 | -0.86 |
| Cysteine, 3TMS | 11.89 | -0.82 |
| L-Arginine, 3TMS | 15.629 | -0.72 |
| Arabinose isomer 1, 4TMS | 17.34 | -0.69 |
| 3-Heptene, 2,2,4,6,6-pentamethyl- | 16.511 | -0.64 |
| Decane, 2,3,5,8-tetramethyl- | 17.86 | -0.64 |
| Propane, 1,3-bis(dimenthyphosphino)- | 14.12 | -0.6 |
| butanamide, N-(3,5-difluoro-2-hydroxy-4-methylphenyl)-4-(3-pentadecylphenoxy)- | 23.905 | -0.58 |
| 3-Amino-2-piperidone, 2TMS derivative | 10.21 | -0.53 |
| Propane, 2-bromo-2-methyl- | 18.086 | -0.52 |
| L-Allo-isoleucine, 2TMS | 7.823 | -0.5 |
| Citric acid, 4TMS | 15.55 | -0.47 |
| Methyl L-alaninate, 2TMS derivative | 11.067 | -0.44 |
| L-Aspartic acid, N-trimethylsilyl-, 1-methyl ester, 4-trimethylsilyl ester (isomer 2) | 9.974 | -0.42 |
| Timonacic, 2TMS derivative | 15.994 | -0.39 |
| 8-Methoxy-2-(p-methoxyphenyl)-1,2,4,5-tetrahydro-1-benzazocine-3,6-dione | 15.08 | -0.35 |
| 1-Nonene, 4,6,8-trimethyl- | 19.041 | -0.21 |
| Ethanolamine, 3TMS | 6.015 | -0.18 |
| tert-Butyldimethylsilyl 2,3-dimethylbenzoate | 10.815 | -0.16 |
| Silane, tetramethyl- | 16.045 | -0.12 |
| Octane, 2-iodo- | 22.341 | -0.11 |
| Pyroglutamic acid, 2TMS | 11.165 | -0.04 |
| Oxalic acid, allyl decyl ester | 7.974 | -0.03 |
| 2H-1,4-Oxazinimine, 3,4,5,6-tetrahydro-3,3,4,5,5-pentamethyl-N-(2,4,6-trinitrophenyl)- | 11.72 | -0.03 |
| Piperazine, 1-ethyl-4-(4-piperidyl)- | 24.414 | -0.03 |
| 2-naphthalenecarboxamide, N-hexadecyl-1-hydroxy- | 13.39 | -0.01 |
| Heptadecane, 2,6-dimethyl- | 20.94 | -0.01 |
| Silanol, trimethyl-, phosphate (3:1) | 7.438 | 0 |
| 1-Octanol, 2-butyl- | 12.617 | 0 |
| Decane, 5-ethyl-5-methyl- | 23.453 | 0 |
| Oxalic acid, allyl octadecyl ester | 23.937 | 0 |
| Nonane | 6.369 | 0.01 |
| Palmitic acid, 1TMS | 18.385 | 0.01 |
| Fladrafinil | 7.059 | 0.02 |
| Pentane, 2,2,3,4-tetramethyl- | 11.144 | 0.02 |
| Terephthalic acid, di(2-methylphenyl) ester | 9.754 | 0.06 |
| Silane, dimethyl(dimethyl(3-phenylpro-2-enyloxy)silyloxy)(3-phenylpro-2-enyloxy)- | 6.443 | 0.09 |
| Butane-1,3-diol, 1-methylene-3-methyl-, bis(trimethylsilyl)ether | 21.145 | 0.09 |
| Silane, [(dimethylsilyl)methyl]trimethyl- | 24.496 | 0.09 |
| 1,5-Octadiene, 7-methyl-3-(1-methylethyl)- | 13.912 | 0.13 |
| Pyrrolo[1,2-a]quinoline-1-ethanol, dodecahydro-6-(2,4-pentadienyl)-, [1R-[1α,3aβ,5aα,6α(Z),9aα]]- | 8.019 | 0.14 |
| Carbonic acid, eicosyl vinyl ester | 27.423 | 0.14 |
| 5-(2-Aminoethyl)thiophene-2-sulfonamide, N,N,N',N'-tetrakis(trimethylsilyl)- | 6.786 | 0.15 |
| Pinacol, 2TMS derivative | 23.558 | 0.15 |
| Butylated Hydroxytoluene | 11.191 | 0.17 |
| Cyclohexane, 2,4-diisopropyl-1,1-dimethyl- | 13.92 | 0.17 |
| 1-(2-Methoxyethoxy)-2-methyl-2-propanol, TMS derivative | 22.465 | 0.17 |
| N-(2-Nitrophenyl)ethylenediamine, 2TMS derivative | 14.309 | 0.22 |
| Benzoic acid, 1TMS | 7.09 | 0.23 |
| 2,2,3,3,4,4,4-Heptafluoro-N-[[4-[(2,2,3,3,4,4,4-heptafluorobutanoylamino)methyl]phenyl]methyl]butanamide | 8.285 | 0.25 |
| L-Cystine, 4TMS | 20.841 | 0.29 |
| 3-Hydroxy-N-(1-hydroxy-4-methylpentan-2-yl)-5-oxo-6-phenylhexanamide, 3TMS | 11.665 | 0.3 |
| Pyruvic acid, 2TMS | 10.412 | 0.32 |
| 2,4,6-Tris(1,1-dimethylethyl)-4-methylcyclohexa-2,5-dien-1-one | 13.311 | 0.33 |
| 1-Hexyl-2-nitrocyclohexane | 6.031 | 0.36 |
| Phenol, 2,6-bis(1,1-dimethylethyl)-4-methyl-, methylcarbamate | 13.51 | 0.45 |
| Proline, 2TMS | 7.872 | 0.67 |
| L-Histidine, 3TMS | 16.559 | 0.68 |
| 1,2-Ethenediol, 2TMS derivative | 23.875 | 0.68 |
| N-α-Acetyl-L-Lysine, 3TMS derivative | 15.88 | 0.74 |
| Furethidine | 11.612 | 0.79 |
| Melibiose isomer 1, 8TMS | 25.327 | 0.89 |
| L-Valine, 2TMS | 6.535 | 1.72 |
| DL-Threonine, 3TMS | 9.315 | 1.89 |
| L-Phenylalanine, 2TMS | 12.674 | 3.17 |
| D-Chiro-Inositol, 6TMS | 18.111 | 3.56 |
| L-Glutamic acid, 3TMS | 12.672 | 5.21 |

**Table S4**: Significant results and t-test statistics obtained for the LW and LA mean’s comparison of the bacterial communities reads. *Df =* degrees of freedom*, p =* probability.

|  | *t-value* | Df | *p* |
| --- | --- | --- | --- |
| Order Rhodobacterales | -11.3 | 12 | < 0.0001 |
| Order Caulobacterales | 12.75 | 12 | < 0.0001 |
| Family *Hyphomonadacea* | 24.6 | 6 | < 0.0001 |
| *Marinobacter-a (LA vs Transplanted)* | 9.23 | 6 | <0.0001 |
